# Supplementary material for: Multi‐dataset identification of innovative feature genes and molecular mechanisms in keratoconus
Source: J Cell Mol Med. 2024 Sep 19;28(18):e70079. doi: 10.1111/jcmm.70079 (PMC11412914; doi:10.1111/jcmm.70079)
Supplement: Supplementary file 2 — Material S2. [file JCMM-28-e70079-s002.zip › 7.GSEA analysis/1.ARL11/KEGG_PROSTATE_CANCER.html]

Details for gene set KEGG\_PROSTATE\_CANCER[GSEA]

|  || Dataset | ARL11.group.cls#h\_versus\_l |
| Phenotype | group.cls#h\_versus\_l |
| Upregulated in class | l |
| GeneSet | KEGG\_PROSTATE\_CANCER |
| Enrichment Score (ES) | -0.23034981 |
| Normalized Enrichment Score (NES) | -0.9850415 |
| Nominal p-value | 0.4832347 |
| FDR q-value | 1.0 |
| FWER p-Value | 1.0 |
Table: GSEA Results Summary

  

Fig 1: Enrichment plot: KEGG\_PROSTATE\_CANCER      
 Profile of the Running ES Score & Positions of GeneSet Members on the Rank Ordered List

  

| SYMBOL | TITLE | RANK IN GENE LIST | RANK METRIC SCORE | RUNNING ES | CORE ENRICHMENT || 1 | PIK3R3 | na | 2180 | 0.284 | -0.0282 | No |
| 2 | CCNE2 | na | 2488 | 0.266 | -0.0045 | No |
| 3 | PIK3CG | na | 2870 | 0.247 | 0.0146 | No |
| 4 | SRD5A2 | na | 3151 | 0.234 | 0.0352 | No |
| 5 | INSRR | na | 4553 | 0.181 | 0.0168 | No |
| 6 | IGF1R | na | 5874 | 0.140 | -0.0041 | No |
| 7 | PDGFRA | na | 5971 | 0.138 | 0.0099 | No |
| 8 | PIK3R1 | na | 6079 | 0.135 | 0.0234 | No |
| 9 | SOS1 | na | 6194 | 0.132 | 0.0363 | No |
| 10 | AR | na | 6920 | 0.114 | 0.0292 | No |
| 11 | PDGFA | na | 7165 | 0.108 | 0.0354 | No |
| 12 | CREBBP | na | 7701 | 0.098 | 0.0319 | No |
| 13 | IKBKB | na | 8698 | 0.079 | 0.0129 | No |
| 14 | PIK3R2 | na | 8783 | 0.078 | 0.0200 | No |
| 15 | MTOR | na | 8802 | 0.078 | 0.0290 | No |
| 16 | AKT2 | na | 9348 | 0.070 | 0.0218 | No |
| 17 | TP53 | na | 9447 | 0.068 | 0.0273 | No |
| 18 | INS | na | 9462 | 0.068 | 0.0352 | No |
| 19 | SOS2 | na | 9955 | 0.061 | 0.0284 | No |
| 20 | PIK3R5 | na | 10118 | 0.059 | 0.0310 | No |
| 21 | BRAF | na | 10628 | 0.052 | 0.0226 | No |
| 22 | KRAS | na | 10630 | 0.052 | 0.0290 | No |
| 23 | MDM2 | na | 10671 | 0.052 | 0.0341 | No |
| 24 | PDPK1 | na | 10673 | 0.052 | 0.0404 | No |
| 25 | FOXO1 | na | 10691 | 0.051 | 0.0462 | No |
| 26 | TCF7L2 | na | 10815 | 0.050 | 0.0487 | No |
| 27 | EGFR | na | 11115 | 0.046 | 0.0457 | No |
| 28 | HSP90AA1 | na | 11158 | 0.046 | 0.0501 | No |
| 29 | FGFR2 | na | 11489 | 0.042 | 0.0457 | No |
| 30 | CREB3L1 | na | 12911 | 0.028 | 0.0081 | No |
| 31 | PIK3CA | na | 13512 | 0.023 | -0.0065 | No |
| 32 | EGF | na | 13590 | 0.022 | -0.0060 | No |
| 33 | PTEN | na | 14202 | 0.017 | -0.0215 | No |
| 34 | CTNNB1 | na | 14653 | 0.014 | -0.0328 | No |
| 35 | HSP90AB1 | na | 14766 | 0.013 | -0.0344 | No |
| 36 | ATF4 | na | 14783 | 0.013 | -0.0333 | No |
| 37 | CCNE1 | na | 14811 | 0.013 | -0.0326 | No |
| 38 | CHUK | na | 15127 | 0.010 | -0.0404 | No |
| 39 | ERBB2 | na | 16056 | 0.003 | -0.0668 | No |
| 40 | RB1 | na | 16201 | 0.002 | -0.0707 | No |
| 41 | E2F2 | na | 16509 | 0.000 | -0.0795 | No |
| 42 | CCND1 | na | 16546 | -0.000 | -0.0805 | No |
| 43 | MAP2K2 | na | 16997 | -0.003 | -0.0931 | No |
| 44 | GSTP1 | na | 17007 | -0.003 | -0.0930 | No |
| 45 | CREB3L4 | na | 17310 | -0.005 | -0.1011 | No |
| 46 | E2F1 | na | 17721 | -0.008 | -0.1119 | No |
| 47 | AKT1 | na | 18085 | -0.011 | -0.1210 | No |
| 48 | TCF7 | na | 18185 | -0.012 | -0.1225 | No |
| 49 | IGF1 | na | 18329 | -0.013 | -0.1251 | No |
| 50 | HRAS | na | 18435 | -0.013 | -0.1265 | No |
| 51 | BAD | na | 19342 | -0.019 | -0.1502 | No |
| 52 | CDKN1B | na | 20317 | -0.026 | -0.1752 | No |
| 53 | CASP9 | na | 20358 | -0.026 | -0.1731 | No |
| 54 | GSK3B | na | 20728 | -0.029 | -0.1802 | No |
| 55 | CREB3 | na | 20880 | -0.030 | -0.1808 | No |
| 56 | HSP90B1 | na | 21584 | -0.036 | -0.1967 | No |
| 57 | MAPK3 | na | 22614 | -0.046 | -0.2208 | No |
| 58 | IKBKG | na | 22799 | -0.048 | -0.2202 | No |
| 59 | GRB2 | na | 23043 | -0.051 | -0.2211 | No |
| 60 | CREB3L2 | na | 23209 | -0.052 | -0.2194 | No |
| 61 | RAF1 | na | 23336 | -0.054 | -0.2165 | No |
| 62 | PIK3CB | na | 23818 | -0.060 | -0.2230 | Yes |
| 63 | EP300 | na | 23862 | -0.060 | -0.2169 | Yes |
| 64 | BCL2 | na | 24069 | -0.063 | -0.2151 | Yes |
| 65 | NFKBIA | na | 24454 | -0.069 | -0.2178 | Yes |
| 66 | TCF7L1 | na | 24530 | -0.070 | -0.2114 | Yes |
| 67 | MAPK1 | na | 24626 | -0.071 | -0.2054 | Yes |
| 68 | E2F3 | na | 25055 | -0.077 | -0.2084 | Yes |
| 69 | PIK3CD | na | 25759 | -0.087 | -0.2181 | Yes |
| 70 | PDGFD | na | 25857 | -0.088 | -0.2102 | Yes |
| 71 | CDK2 | na | 25887 | -0.088 | -0.2002 | Yes |
| 72 | ARAF | na | 26195 | -0.093 | -0.1976 | Yes |
| 73 | NFKB1 | na | 26767 | -0.104 | -0.2015 | Yes |
| 74 | CREB1 | na | 27227 | -0.113 | -0.2009 | Yes |
| 75 | TGFA | na | 27293 | -0.114 | -0.1888 | Yes |
| 76 | CDKN1A | na | 27494 | -0.119 | -0.1800 | Yes |
| 77 | AKT3 | na | 27547 | -0.120 | -0.1669 | Yes |
| 78 | PDGFC | na | 27761 | -0.125 | -0.1577 | Yes |
| 79 | KLK3 | na | 27780 | -0.125 | -0.1429 | Yes |
| 80 | NRAS | na | 27790 | -0.126 | -0.1278 | Yes |
| 81 | CREB3L3 | na | 27885 | -0.128 | -0.1149 | Yes |
| 82 | RELA | na | 29186 | -0.162 | -0.1326 | Yes |
| 83 | PDGFRB | na | 29523 | -0.173 | -0.1212 | Yes |
| 84 | MAP2K1 | na | 31070 | -0.223 | -0.1386 | Yes |
| 85 | PDGFB | na | 32445 | -0.283 | -0.1436 | Yes |
| 86 | LEF1 | na | 32575 | -0.292 | -0.1115 | Yes |
| 87 | CREB5 | na | 34086 | -0.433 | -0.1022 | Yes |
| 88 | NKX3-1 | na | 34313 | -0.477 | -0.0504 | Yes |
| 89 | FGFR1 | na | 34437 | -0.510 | 0.0084 | Yes |
Table: GSEA details [plain text format]

  

Fig 2: KEGG\_PROSTATE\_CANCER      
 Blue-Pink O' Gram in the Space of the Analyzed GeneSet

  

Fig 3: KEGG\_PROSTATE\_CANCER: Random ES distribution      
 Gene set null distribution of ES for **KEGG\_PROSTATE\_CANCER**
